# Comprehensive Ecological Risk Assessment of Heavy Metals Based on Species Sensitivity Distribution in Aquatic of Coastal Areas in Hong Kong

**DOI:** 10.3390/ijerph192013376

**Published:** 2022-10-17

**Authors:** Shaowei Rong, Jin Wu, Xiaoyuan Cao, Yue Sun

**Affiliations:** 1 College of Architecture and Civil Engineering, Beijing University of Technology, Beijing 100124, China; 2 Faculty of Geographical Science, Beijing Normal University, No. 19 Xinjiekouwai Street, Haidian District, Beijing 100875, China; 3 Marine Consulting Center, Ministry of Natural Resources, Maguanying Fengtai District, Beijing 100071, China

**Keywords:** species sensitivity distribution, ecotoxicology, environmental toxicology, ecological risk assessment, heavy metals, marine pollution

## Abstract

In recent decades, the ecological environment of some coastal areas in China has been seriously affected by terrestrial pollutants, and there is an urgent need for ecological risk assessment of China’s coastal environment. The assessment of heavy metal pollution in Hong Kong waters was carried out using different environmental and ecological indicators. The heavy metal contents (Cu, Pb, Zn, Cd, As, Cr, and Hg) in the near coast of Hong Kong were analyzed for two different seasons of the year 2018 (April—spring and September—autumn). We assessed the distribution and enrichment of heavy metals in the near coast of Hong Kong, and the potential biohazardous effects were assessed using the species sensitivity distribution method. The results showed that only Pb, Zn, and Hg in seawater exceeded the Class I standard. Pb, Zn, Cd, and As in organisms exceeded the standard, and no heavy metals exceeded the standard in sediments. The species sensitivity distribution method indicated that the biohazardous factor of heavy metals of the Hong Kong coast is higher in spring than in autumn, and the potential hazard ratio has the characteristics of high northwest and low southeast, which leads to its msPAF also having these characteristics. From the correlational analyses among heavy metals, we found that the pH change in seawater was related to the concentration of heavy metals, the concentration of heavy metals in seawater was proportional to the salinity of seawater, Pb and Cu were likely to have the same source, and Zn and Cd may not have the same emission sources as the other heavy metals. Overall, heavy metal contamination of seawater, sediments, and organisms near the Hong Kong coast was within acceptable limits, but the problem of heavy metal dispersion should be prevented.

## 1. Introduction

The marine ecosystem is an important part of the biosphere, and coastal and estuarine areas are important habitats for human beings. They not only provide abundant fishery resources for human beings, but also regulate the climate through gas and marine thermal regulation [1]. With the development of science, technology, and industry, increasing levels of heavy metals are artificially discharged and continuously accumulate in the marine system [2,3]. As one of the most serious pollutants in the ecological environment, heavy metals are characterized by high toxicity, strong persistence, bioaccumulation, and non-biodegradability [4,5,6]. Heavy metal pollution not only reduces the quality of seawater and aquatic products, but also accumulates in important organs, such as the kidney, bones, and liver, which pose a threat to human health [6,7,8]. Therefore, it is necessary to study the enrichment of heavy metals in the marine system, especially in seawater, sediments, and organisms near the coast, to determine the potential ecological risks and future development of the marine system.

The problems of rapid population growth, industrialization, and urbanization in recent decades have resulted in significant increases in freshwater use and wastewater production, resulting in increasingly poor water quality. Globally, major environmental and economic problems caused by water quality issues abound. The importance of protecting water ecology is has been indicated by the United Nations Sustainable Development Goals (SDGs) in 2015, under no. 6, “Clean Water and Sanitation”, and no. 14, “Life Below Water” [9].

Ecological risk assessment refers to the assessment of factors that could reduce the health, productivity, genetic structure, economic value, and aesthetic value of a species, population, or ecosystem within the system [10]. Simply speaking, it refers to the possibility of assessing the adverse ecological consequences of ecosystems affected by one or more stress factors [11]. Therefore, ecological risk assessment is an important means to quantify the ecological harm of toxic pollutants. The goal is to obtain a concentration threshold or risk value, and provide a reference for environmental decision-making or the formulation of relevant standards or benchmarks. In recent decades, the research scale of ecological risk assessments has expanded from a single population to an ecosystem, and the research object has also expanded from terrestrial ecosystems to marine ecosystems. Species sensitivity distribution (SSD) is an important method to derive standard values of water quality, which originated in the United States and Europe in the late 1970s. It has played a vital role in ecological risk assessment and water environment standard derivation or standard formulation [12,13,14].

Since China’s reforms and borders opening up in 1978, coastal cities in Guangdong have rapidly developed, resulting in many areas facing challenges of heavy metal pollution. Many studies have shown that many coastal areas in China have been seriously polluted by heavy metals [15,16]. Pan and Wang (2012) showed that heavy metal pollution along the coastline changes with local economic development, pollution sources, and geographical conditions [17]. Although heavy metal pollution is better in the coastal areas of southern China than in the north, Hong Kong, a traditional industrial area, has been classified as a ‘hot spot’ because of its severe heavy metal pollution [17,18]. Wong et al. found that the rapid economic development in the coastal areas of southern China since opening its borders in 1978 has brought with it significant environmental side effects, particularly in the local electronics industry, which has produced significant heavy metal pollution [18]. At present, most studies on Hong Kong and abroad have focused on the ecotoxicological properties and bioaccumulation of heavy metals in sediments of the Hong Kong sea area [15,19,20,21]. Few studies have been conducted on heavy metal pollution in coastal seawater, sediments, and organisms in Hong Kong. Therefore, based on SSD ecological risk assessment, this study sampled and investigated seawater, sediments, organisms, and productivity levels near the coast of Hong Kong.

As an important part of the Guangdong-Hong Kong-Macao Greater Bay Area, Hong Kong has a superior geographical position. However, due to the old industrial area along the coast of Guangdong, some sea areas are seriously polluted by heavy metals [22,23]. Therefore, it is necessary to assess the ecological risk of marine heavy metal pollution in Hong Kong. In this study, the heavy metal pollution levels of in seawater, sediments, and marine organisms were evaluated. We assessed the contamination levels based on the SSD curves of heavy metals, the 5% hazard concentration (HC5) of marine organisms at different trophic levels, and the potential impact ratio (PAF), providing a basis for ecological risk assessment and risk management of heavy metal pollution in the marine environment of the South China Sea.

## 2. Materials and Methods

### 2.1. Study Area

**Figure 1:**
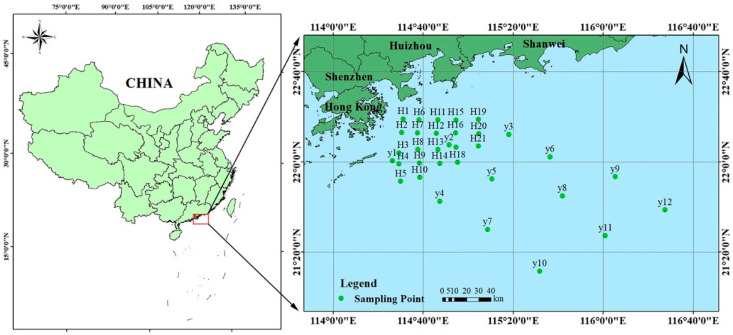
Study area and sampling sites.

Hong Kong is located in the south of China (114°15′ E to 116°35′ E, 22°15′ N to 22°30′ N) and east of the Pearl River Estuary. The region covers 262 islands, including Hong Kong Island, Kowloon, New Territories, and surrounding islands. The total land area is 1106.66 square kilometers and the sea area is 1648.69 square kilometers (Figure 1). It is one of the regions with the highest population density in the world. Hong Kong has a marine subtropical monsoon climate with four distinct seasons. The annual average temperature is 23.3 °C, and rainfall is concentrated from May to September, accounting for about 80% of the annual rainfall. Hong Kong is adjacent to the continental shelf, with a vast ocean surface and numerous islands. The fishery production environment is unique, and more than 150 kinds of marine fish with commercial value are produced.

The Hong Kong region is rich in runoff and has well-developed surface water systems. However, the water system has a limited role and there are no large rivers. Apart from the Shenzhen River, which is the boundary river between Hong Kong and Shenzhen, the main rivers are Shing Mun River, Indus River, Lam Tsuen River, Yuen Long River, and Kam Tin River, the majority of which are no more than 5 miles long. Situated near the Pearl River Estuary, the bay created by the movement of ocean currents also gives Hong Kong a reputation as a deep-water port. Moreover, the ocean currents flow from east to west in the Pearl River Estuary, and their cleaning capacity protects the aquatic ecological environment of Hong Kong to a certain extent, and transports pollutants to other regions.

A total of 21 survey stations were laid out in the offshore section of Hong Kong, with a large survey station spacing of about 10 km. On-site sampling was made for two different seasons of the year 2018 (April—spring and September—autumn). All stations were surveyed for seawater quality, 15 stations for sediment and marine life (including chlorophyll a content, primary productivity, phytoplankton, zooplankton, and benthic organisms) surveys, and 12 stations for marine biological quality (consistent with fishery resource survey stations) surveys (Figure 1).

### 2.2. Sample Collection and Analysis

#### 2.2.1. Sample Collection

Sample collection, processing, and analysis were carried out in accordance with the national and industrial standards of the People’s Republic of China, specifically ‘The specification for marine monitoring’ (GB 17378-2007), ‘Specifications for oceanographic survey’ (GB/T 12763-2007), and ‘Code of practice for marine monitoring technology’ (HY/T 147-2013).

A sample of 300 mL of seawater was collected at 1 m below the water surface, 200 mL of which was filtered through a 0.45 μm, Ø60 mm microporous membrane. Nitric acid was added to each seawater sample to adjust the pH to <2 and then divided into two cryogenically frozen portions for the determination of heavy metals (As, Cd, Pb, Cu, Hg, Cr, and Zn) in seawater. The remaining 100 mL of seawater was filtered through a 0.45–0.7 μm, Ø47 mm glass fiber filter and then stored in a dry cooler and brought back to the laboratory for spectrophotometric analysis to determine chlorophyll a content [24].

Sediment samples were collected using a 0.05 m^2^ grab mud collector, collected 0–5 cm from the surface layer of the seafloor sediment, and stored in polyethylene bags, kept refrigerated and protected from light. After removing rocks and other debris in the laboratory, the sediment samples for the determination of As, Cd, Pb, Cu, Cr, and Zn were dried in an oven at 105 °C for approximately 72 h and sediment samples for the determination of Hg were naturally dried at room temperature. Samples were ground after drying and then sieved through a 160 mesh nylon sieve for As, Cd, Pb, Cu, Cr, and Zn, and through an 80 mesh nylon sieve for Hg.

Marine organisms were sampled near the site using a trawl or grab sampler. A total of 26 samples of two species of fish, four crustaceans, and one shellfish organism were collected at 12 sites during the spring survey and a total of 23 samples of four species of fish, four crustaceans, and one shellfish organism were collected during the autumn survey. Heads, tails, and guts were removed from fish; crustaceans were decapitated and de-shelled; and mollusks were de-shelled. Remaining muscle tissue (approximately 100 g) of fish and crustaceans and edible parts of mollusks were cleaned and weighed. The organism samples were freeze-dried for 24 h in the laboratory and then crushed and digested in concentrated HNO_3_ for heavy metal analysis.

#### 2.2.2. Laboratory Analyses

Two methods were used for the determination of heavy metals, atomic fluorescence spectrometer (AFS) for As and Hg and atomic absorption spectrometry for Cu, Pb, Zn, Cr, and Cd.

Heavy metals in seawater were analyzed directly without enrichment. The concentration of As and Hg were analyzed by AFS (AFS-930); the concentration of Cu, Pb, Cr, and Cd were determined by graphite furnace atomic absorption spectroscopy (GFASS; iCE3500 atomic absorption spectrophotometer); and Zn was determined using flame atomic absorption spectroscopy (FASS; iCE3500 atomic absorption spectrophotometer) [25]. The seawater samples used to measure chlorophyll a concentration were extracted with a 90% acetone solution of phytoplankton pigments and the absorbance values were measured at 664, 647, 630, and 750 nm, in that order.

The 0.5 g sediment samples used for the determination of Cu, Pb, Zn, Cr, and Cd were accurately weighed and placed in a sample cup with a small amount of water, and 10 mL HNO_3_ and 5 mL HF were added to fully mix the acid with the sample. The sample cup was placed in a high-pressure digestion tank for microwave digestion. After cooling, the sample cup was taken out and placed on an electric heating plate at 160 °C. After evaporation until nearly dry, it was transferred to a 25 mL volumetric flask and diluted with pure water. The constant volume sample was placed in an iCE3500 atomic absorption spectrophotometer to analyze the content of heavy metals in the sample (the concentration of Zn was measured by FASS; concentrations of Cu, Pb, Cr, and Cd were measured using GFASS). The sediment samples for the determination of Hg and As were accurately weighed to 0.5 g and placed in a sample cup moistened with a small amount of water, then 3 mL concentrated HNO_3_ and 7 mL H_2_O was added and fully mixed. The sample cup was placed in a high-pressure digestion tank for microwave digestion, taken out after cooling, then the sample was transferred to a 25 mL volumetric flask with pure water to constant volume and analyzed using AFS (AFS-930).

Accurately weighed 0.5 g biologically dried samples for measuring Hg concentration were placed in a 50 mL beaker, 10 mL nitric acid and 1 ml perchloric acid were added, then it was covered by the watch glass and placed overnight. The next day, the sample was placed on a 160 °C electric heating plate until it no longer produced yellowish-brown smoke. After cooling to room temperature, we added 5 mL of hydrochloric acid, transferred the sample to a 50 mL volumetric flask with 1% oxalic acid solution diluted for later use. An accurate weighing of 0.5 g of the biological dry sample was placed in a 50 mL volumetric flask for As measurement, 10 mL of nitric acid was added, and the watch glass was covered and left to stand overnight. The next day, the sample was placed on a 160 °C electric heating plate for heating and digestion, and nitric acid was repeatedly added until the solution became colorless. Then 1 mL perchloric acid was added for heating and digestion of the remaining small amount of solution. After cooling, the sample was transferred to a 50 mL volumetric flask, 25 mL sulfuric acid solution, and 5 mL thiourea–ascorbic acid reducing agent were added and diluted with water to the calibration line. Hg and As concentrations were measured using AFS (AFS-930). The 0.1 g biologically dried samples used for the determination of Cu, Pb, Zn, Cr, and Cd were accurately weighed. The samples were placed in a 50 mL beaker and wet with water. After 2 mL of nitric acid was added, we covered the watch glass, placed the sample on the electric heating plate, and heated at a low temperature until the foam basically disappeared. Then 0.5 mL of hydrogen peroxide was slowly added. After the watch glass was covered, it was heated on the electric heating plate at 160 °C for 20 min. Then 1.5 mL of hydrogen peroxide was added to continue heating and evaporation to 1 ml. After adding 1 mL of nitric acid and 1.5 mL of hydrogen peroxide, the sample was heated and evaporated to 0.5 mL. The sample for measuring Cr had 1 mL ascorbic acid solution added before constant volume. The samples were put into the iCE3500 atomic absorption spectrophotometer to analyze the content of heavy metals in the samples (GFASS was used to measure the concentration of Cu, Pb, Cd, and Cr; measurement of Zn concentration used FASS).

All analytical data were subject to strict quality control. The instrument was calibrated daily to the calibration standard. Accuracy and precision were verified using certified reference materials from the State Bureau of Oceanic Administration (GB 17378.4-2007 for seawater; GB 17378.5-2007 for sediments; GB 17378.6-2007 for organisms), the specific detection limit and relative standard deviation are shown in Table S1. Data processing and quality control of the analysis was carried out according to the certified reference material from State Bureau of Oceanic Administration (GB 17378.2-2007) and the analysis yielded recoveries of heavy metals that varied between 86 and 95%. The difference in metal concentrations between the study results and certified values was generally <10% [26].

### 2.3. Data Analysis

Pearson correlation analysis, non-metric multidimensional scaling analysis (NMDS), Adonis test, Procrustes analysis, and constraint coordination principal coordinate analysis (CPCOA) were used to study the correlations or differences between heavy metals and samples. The coexistence of heavy metals and organisms in the ocean was explored by network analysis based on its strong and significant correlation matrix (*p* = 0.8). Gephi (v0.9.2) was used for network visualization. Statistical analysis was performed using several packages in R (v3.4.2), including gunifrac, car, and vegan. The Kriging interpolation method of ArcGIS (v10.8, ESRI, Redlands, CA, USA) analyzed the distribution characteristics of heavy metals in sediments.

Species sensitivity analysis was performed using the National Ecological Environment Criteria Calculation Software-Species Sensitivity Distribution Method (EEC-SSD) developed by the Chinese Academy of Environmental Sciences. The toxicity data used in species sensitivity analysis were from the EPA ECOTOX database (http://www.epa.gov/ecotox/, accessed on 7 June 2022), the screening endpoint was EC50 and LC50, and the medium was acute (≤10 d) data of seawater.

### 2.4. Flowability of Heavy Metals in Seawater and Sediments

Adsorption capacity of heavy metals from liquid (seawater) to solid (sediment) is expressed by the distribution coefficient (*K_d_*) between sediment and water [27,28,29].

(1) $$K_{d}=\frac{C_{sed}}{C_{sea}}$$

*C_sed_* and *C_sea_* represent concentrations of target heavy metals in sediments and seawater, respectively. A high *K_d_* value indicates that the metal is preferentially retained by sediments, whereas a low value indicates that the metal is mainly retained in water [27].

### 2.5. Seawater Pollution Assessment

There are many evaluation methods for water quality, mainly an index system and fuzzy mathematics. The single water quality evaluation in this study adopted the single standard index method currently used in China. The single pollution index method is to analyze and evaluate the single index item by item through the evaluation criteria. Through the index calculation, the largest category of each factor is selected as the overall evaluation result of the sample. The comprehensive evaluation of water quality adopted the comprehensive index WQI method [30].

(2) $$A_{i}=C_{i}/C_{si}$$

(3) $$\mathrm{WQI}=\frac{1}{n}\overset{n}{\underset{i=1}{\sum }}A_{i}$$

*A_i_* is the standard index for *i* pollutants; *C_i_* is the measured concentration of pollutant *i*; and *C_si_* is the evaluation standard of *i* pollutants. Evaluation of the implementation of the ‘People’s Republic of China Seawater Quality Standards’ (GB3097-1997) in the first class of standards, used two, three, and four types of standards as a reference. WQI is a comprehensive water quality index, *A_i_* is a single factor standard index, and n is the number of items involved in the evaluation of all water quality parameters. A WQI < 1 indicated cleanliness; 1 < WQI ≤ 2 represented light pollution; 2 < WQI ≤ 3 was moderate pollution; and WQI > 3 indicated serious pollution.

### 2.6. Biological Quality Assessment

A primary productivity and diversity index were used to evaluate biological quality. The CADEE (1975) formula was used to estimate primary productivity according to chlorophyll a, transparency, water depth, illumination time, and the carbon assimilation coefficient [31]. The Shannon–Wiener index was used to measure the diversity index.

(4) $$P=C_{a}QLt/2$$

(5) $$H^{\prime }=-\overset{S}{\underset{i=1}{\sum }}P_{i}log_{2}P_{i}$$

(6) $$Chla=11.85\times \left(E_{664}-E_{750}\right)-1.54\times \left(E_{647}-E_{750}\right)-0.08\times \left(E_{630}-E_{750}\right)$$

*P* is primary productivity, mg·C/m^2^·d. *C_a_* is the surface chlorophyll a concentration, mg/m^3^. *Q* is the assimilation coefficient, mg·C/(mgChl-a·h), which was 3.7 here [32]. *L* is the depth of the photic zone, m, taken as three times the transparency reading, and *t* is the light time, h. *H’* is the species diversity index, *S* is the total number of species in the sample, and *P_i_* is the ratio of the number of the *i*th individual to the total number. *Chla* is the mass concentration of chlorophyll a in the sample, mg/m^3^; *E*_664_ is the absorbance value of the sample at 664 nm wavelength; *E*_647_ is the absorbance value of the sample at 647 nm wavelength; *E*_630_ is the absorbance value of the sample at 630 nm wavelength; and *E*_750_ is the absorbance value of the sample at 750 nm wavelength.

### 2.7. Species Sensitivity Distribution Assessment

In order to use the toxicological data of different species to formulate environmental standards conducive to the protection of the whole ecosystem and understand the harm of pollutants at the ecosystem level, species sensitivity distribution methods based on different species sensitivity to pollutants are widely used [12,33]. This method has the advantages of simplicity and clear ecological significance. The logistic distribution is a two-parameter continuous probability distribution. When the standard deviation is the same, the logistic distribution has a larger peak and a thicker tail compared with the normal distribution [34,35]. A logistical distribution reflects the concept of ‘limited environmental space’ and is closer to the actual growth state of populations in the environment.

(7) $$F\left(x\right)=\frac{e^{\frac{x-\mu }{\sigma }}}{\sigma {\left(1+e^{\frac{x-\mu }{\sigma }}\right)}^{2}}$$

(8) ln(HC5)=μ−1.6234σ

(9) $$msPAF=1-\prod_{i=1}^{n}\left(1-PAF_{i}\right)$$

In the formula, *x* is the toxic value, μg/L or mg/L; *μ* is the mean value of toxicity, μg/L or mg/L; and σ is shape parameters, μg/L or mg/L. *HC*5 is the cumulative concentration when the proportion of harmful species on SSD curve reaches 5%. A smaller *HC*5 means that it represents a more toxic heavy metal. *PAF* is the proportion of harmful species corresponding to each measured concentration on the SSD curve, and its value can be directly obtained by software mapping. *msPAF* is the proportion of harmful species produced by multiple pollutant combinations, which can reflect the combined pollution of multiple pollutants in water.

## 3. Results and Discussion

### 3.1. Characteristic of Heavy Metals in Seawater, Sediment, and Marine Organisms

#### 3.1.1. Contamination Characteristics of Heavy Metals in the Seawater

The single factor standard index of seawater, sediments, and organisms, calculated by the single pollution index method is shown in Table 1. The measured data of heavy metal concentrations in seawater, sediments, and organisms are shown in Tables S2–S4. Table 1 shows that copper, cadmium, arsenic, and chromium in the samples collected at sampling points in the spring were in line with the first class of seawater quality standards of the People’s Republic of China Seawater Quality Standards (GB3097-1997), and the contents of lead and zinc in some water samples exceeded the first class of seawater quality standards. The exceeding rate of lead was 9.5%, and the lead content of the two samples exceeded the standard. The exceeding standard rate of zinc was 19%, and the zinc content of four samples exceeded the standard. The contents of lead, zinc, and mercury in the exceeded samples all met the second type of seawater quality standards. In the samples collected at the sampling points in autumn, copper, cadmium, arsenic, and chromium all met the first type of seawater quality standards. The exceeding factors were lead, zinc, and mercury. The exceeding rate of lead was 14.3%. The lead content of the three samples exceeded the standard. The exceeding rate of zinc was 14.3%, and the zinc content of the three samples exceeded the standard. The exceeding rate of mercury was 33.3%, and the total mercury content of the seven samples exceeded the standard. However, the over-standard rate was not large, and the content of over-standard factors met the second type of seawater quality standards. Overall, the seawater quality in the offshore survey area of Hong Kong was generally good, and the impact of heavy metal pollution was small.

**Figure 2:**
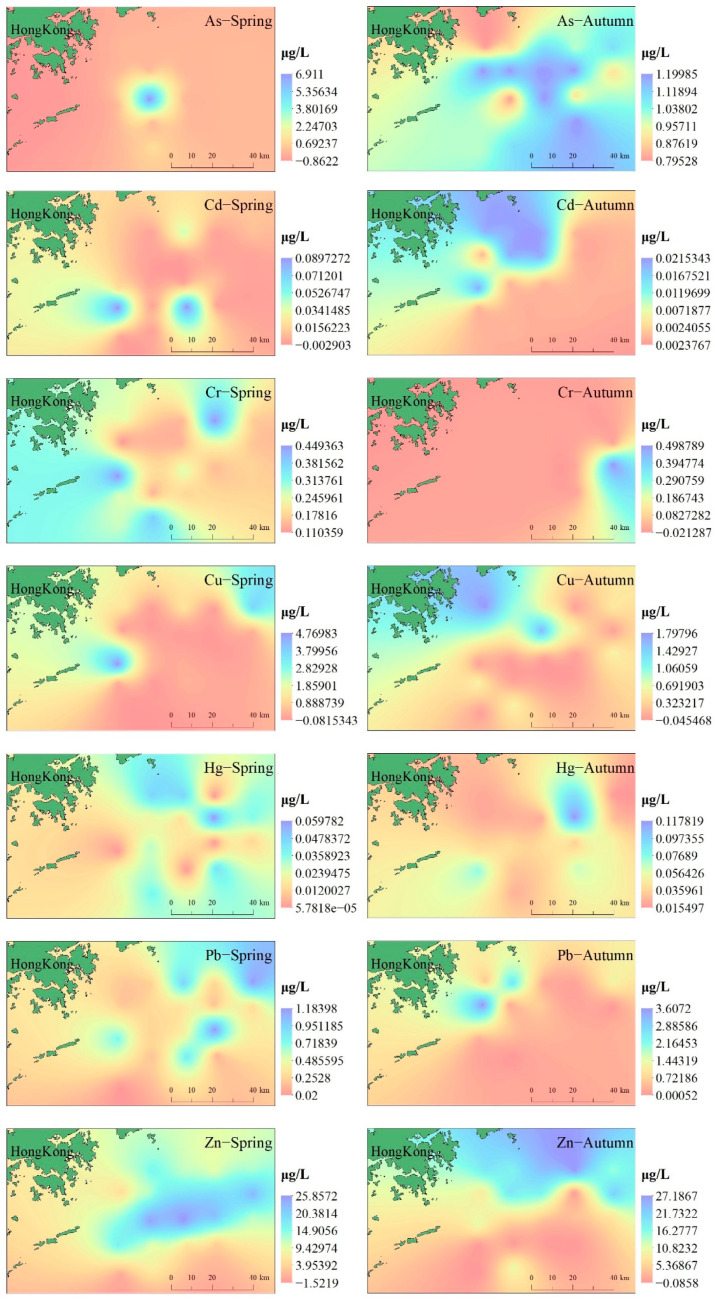
The spatial distribution of heavy metals in seawater.

The Kriging spatial interpolation method was used to simulate the spatial distribution characteristics of heavy metals in the coastal areas of Hong Kong, as shown in Figure 2. As the concentrations of As and Cd in spring seawater samples were not detected much from the concentrations of Cr and Cd in autumn seawater samples, the interpolation results may have some errors. In general, the concentration of heavy metals in the coastal waters of Hong Kong has a seasonal trend, with the concentration of Cu in spring higher than in autumn, but for Pb and Cr, the concentration in autumn is higher than in spring, mainly due to the uneven distribution of seawater temperature caused by the seasonal monsoons in Hong Kong [36]. Except for Hg, As, Cd, Cr, Cu, Pb, and Zn have similar spatial distributions and the maximum concentration is roughly concentrated in the same sea area in the same season. After analyzing the interpolation results of heavy metals, it was found that high concentrations of heavy metals were detected at sites close to the coastline and showed a downward trend towards the ocean, indicating that the Pearl River may also be a main source of heavy metals in the ocean. Combined with the concentration of heavy metals in the sampling points reaching class II water quality standards, we can conclude that the seawater heavy metal pollution in the coastal waters of Hong Kong is still within an acceptable range; however, the diffusion of heavy metals should still be prevented.

**Figure 3:**
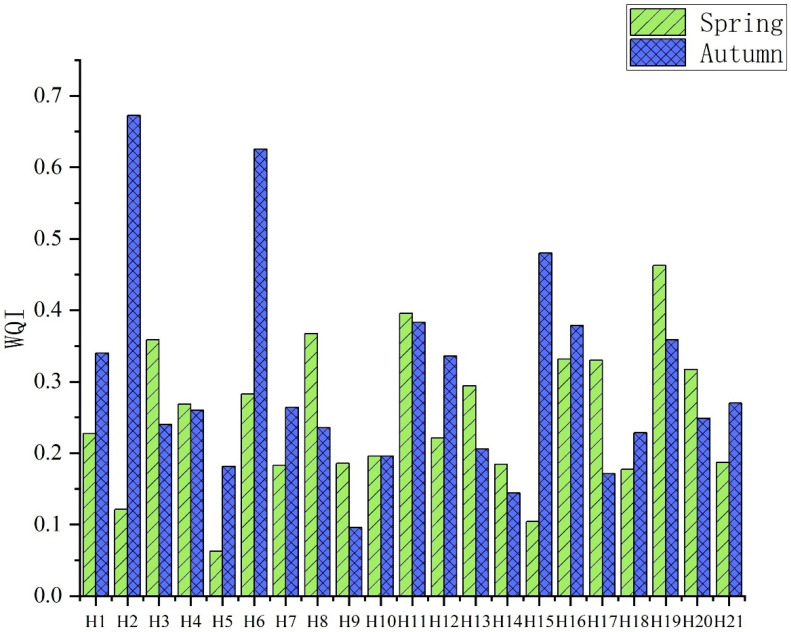
WQI of heavy metals in surface seawater of Hong Kong.

The results of the water quality index (WQI) of heavy metals in the surface seawater of Hong Kong are shown in Figure 3. It can be seen from the chart that the water quality in the west and north of Hong Kong’s offshore coast is better in the spring. That is, the water quality in the offshore coast is better than that of the far off shore, whereas it is the opposite in the autumn. This may be due to seasonal industrial and fishing activities. August is generally the end of the fishing moratorium, and autumn sampling occurred in September, at the peak of fishermen’s fishing activities. Most fishermen on fishing vessels have an arbitrary discharge of domestic sewage, and ballast water or sewage can lead to higher levels of heavy metals, resulting in differences in the marine environment in the autumn compared with spring [37]. At the same time, it can be seen from Figure 3 that the water quality index of the coastal areas of Hong Kong was less than 1, indicating that although there was a certain amount of heavy metal pollution in the coastal areas of Hong Kong, the overall pollution level was considered clean.

#### 3.1.2. Contamination Characteristics of Heavy Metals in Sediments

The results of the sediment quality assessment near the coast of Hong Kong are shown in Table 1. The sediment quality in the investigated sea area was good, and the concentrations of mercury, copper, lead, cadmium, zinc, chromium, and arsenic in the sediments from all stations were in line with the quality standards of the first type of marine sediments. Between the 1960s and 1980s, emissions from PCBs, electroplating, metals, and textile industries in Hong Kong caused serious heavy metal pollution, resulting in heavy metal enrichment in waters and sediments in Hong Kong [38]. In addition, the increase in Cu, Ni, Zn, and Pb contents in sediments was related to the rapid urbanization and industrialization in the 1960s and 1970s [20,37]. The concentrations of heavy metals in sediments along the coast of Hong Kong fell sharply in the 1990s, with the average concentrations of copper and lead falling by 70 and 50%, respectively, as local governments strengthened pollution control and industrial transfer to mainland China [15]. From Table 1, the single pollution index of Hong Kong sediment has a large difference between different points with some spatial variability, and the maximum value generally appears near the coast, which is mainly due to industrial or municipal sewage discharge and marine transportation. Victoria Bay, as the main waterway of Hong Kong, also bears heavy responsibility for discharging sewage, where various heavy metals collect and flow into the sea. This explains why the maximum value of the single pollution index of near-shore sediment generally appears near the coast.

#### 3.1.3. Contamination Characteristics of Heavy Metals in Marine Organisms

The evaluation criteria for the residual toxicity of fish and crustacean samples in marine biological quality were based on the biological quality standards specified in the ‘Brief Regulations for Comprehensive Survey of Coastal and Coast Resources’. The residual toxicity of shellfish samples in marine biological quality was evaluated according to the relevant standards in the national standard, ‘Marine Biological Quality’ (GB 18421-2001) of the People’s Republic of China. The results of biological evaluation by the single factor mass index method are shown in Table 1. The species used to carry out the heavy metal concentration analysis are shown in Table S4.

In spring, 26 marine organisms collected from 12 stations were investigated. Heavy metals such as Pb, Zn, Cd, and As in shellfish collected from multiple stations exceeded the standard, and the exceeding rates of Pb and Zn were 23.08 and 23.08%, respectively. The Cd exceeding standard rate was 26.92%, and As exceeding standard rate was 26.92%; the Cr content of fish organisms from y1, y3, y4, y5, y9, and y12 stations and of crustacean organisms from the y5 station exceeded the standard, and the exceeding rate was 26.92%. Although the exceeding rate of Pb reached 23.08%, the analysis of the raw data showed that the points that exceeded the Class I standard all met the Class II standard, so the pollution of Pb was not considered serious and fell the good range. The concentration of Zn exceeded the Class II standard for one species at only one site. The concentrations of Cd, As and Cr were more serious, but the concentrations of heavy metals in individual species exceeded the national Class II standard at only a few stations. Organisms exceeding the national Class II standard did not occur in marine fisheries waters, mariculture areas, marine nature reserves, or industrial water areas directly related to human consumption. Therefore, the biological quality of the survey area was considered to be good. Exceeding concentrations of Cd and As were found in shellfish organisms, and Cr was found in fish, crustaceans, and shellfish.

Among the 23 marine organisms from the 9 species collected in the autumn survey, there was no excessive heavy metal concentrations. Overall, the biological quality of the surveyed sea area was good, and the pollutant content of shellfish was higher than that of fish and crustaceans.

### 3.2. Marine Organism Quality Assessment

**Figure 4:**
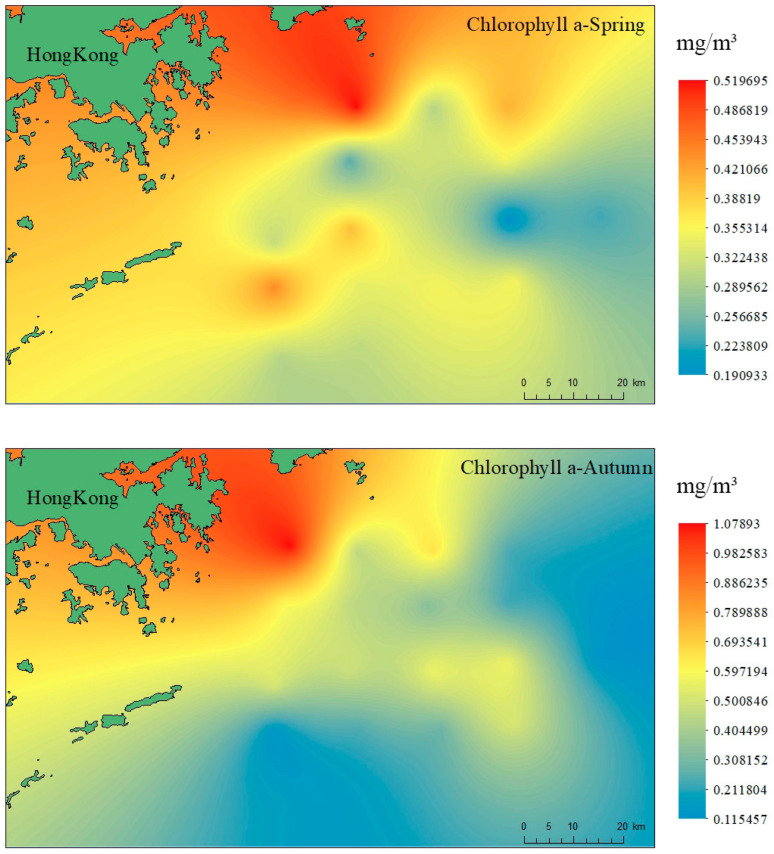
Results of chlorophyll a measurement in coastal region of Hong Kong.

The survey results of marine ecological characteristics near the coast of Hong Kong are shown in Figure 4. According to chlorophyll a concentrations and primary productivity, the average concentration of chlorophyll a in the water column of each station along the Hong Kong coast in spring varied from 0.19–0.52 mg/m^3^, with an average of 0.34 mg/m^3^. According to the biological reference standard (chlorophyll a concentration below 5 mg/m^3^ is considered poor nutrition, 10~20 mg/m^3^ is medium nutrition, more than 30 mg/m^3^ is rich nutrition), the investigation area had poor nutrition levels of chlorophyll a. The variation range of primary productivity was 127–345.7 mg·C/(m^2^·d), with an average of 223.69 mg·C/(m^2^·d). The average concentration of chlorophyll a in the water column of each station in autumn varied from 0.14–1.08 mg/m^3^, with an average of 0.40 mg/m^3^. Chlorophyll a was at poor nutrition levels. The variation range of primary productivity was 101–462.5 mg·C/(m^2^·d), with an average of 239.6 mg·C/(m^2^·d), which was at a medium to low level.

**Figure 5:**
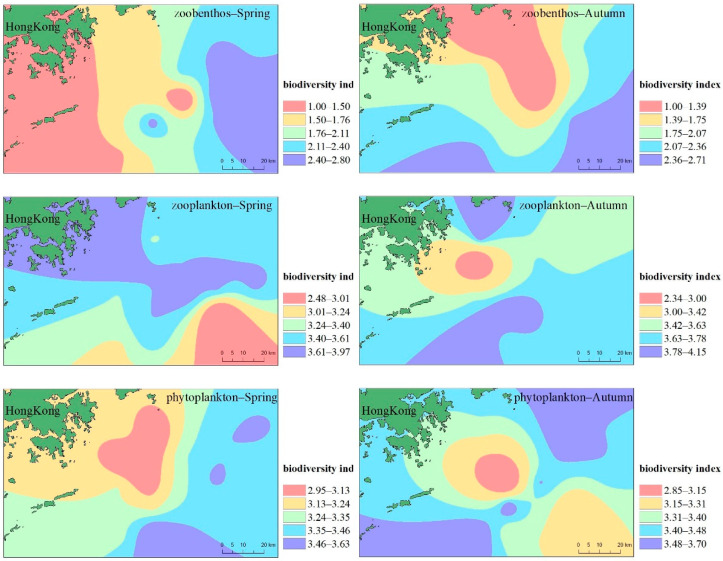
Evaluation results of species diversity index in coast of Hong Kong.

The results of the Hong Kong Coastal Species Diversity Index are shown in Figure 5. The density level of phytoplankton investigated in April 2018 was average, with an average density of 174.472 × 104 cells/m^3^. The number of phytoplankton was dominated by diatoms, with a density of 165.452 × 104 cells/m^3^, accounting for 94.83% of the total density. In the spring survey, the average number of phytoplankton species was 30, and the species diversity index ranged from 2.96 to 3.63, with an average of 3.30. In September 2018, the density level of phytoplankton in autumn was not significantly different from that in spring. The average number of phytoplankton species in the station quadrats in autumn was 32, and the species diversity index was between 2.85 and 3.62, with an average of 3.36, slightly larger than that in spring.

The results of the zooplankton investigation in spring of April 2018 showed that the biomass of zooplankton at each sampling station in this water area was at a medium level, and the distribution was uneven. The change range was 105.00–318.50 mg/m^3^, and the average biomass was 184.83 mg/m^3^. The change range of organism density was 18.85–46.78 ind/m^3^, and the average density was 28.649 ind/m^3^. There were 6 biological groups of zooplankton in the waters surveyed in spring, with a total of 27 species, with an average of 17 species. The average number of individuals in each station was 243, and the species diversity index ranged from 2.48 to 3.97, with an average of 3.53. The highest number of zooplankton appeared at the H13 sampling station. In September 2018, the zooplankton investigated in autumn had 11 biological groups preliminarily identified, with a total of 47 species. The variation range and average biomass of zooplankton biomass were smaller than those in spring, but the variation range and average density of organism density were larger than those in spring. The average number of species of zooplankton at the stations surveyed in autumn were 20; the average number of individuals in each station was 353; the species diversity index ranged from 2.34 to 4.15, with an average of 3.62. In general, the diversity index of zooplankton in the investigated sea area was high. The total number of zooplankton species and individuals in the spring and autumn are shown in Table S5.

A total of 37 species belonging to 31 families and 7 phyla were found in the quantitative survey in spring, among which the number of arthropod species was relatively large, followed by mollusks. The largest composition of biomass was mollusks, accounting for 56.23% of the total biomass, followed by arthropods, annelid and echinoderms. The biomasses of benthic organisms at each station in the spring survey area varied greatly. The highest biomass appeared at station H13, and the lowest biomass appeared at station H8. The highest biomass was 197.22 times that of the lowest biomass. The spring survey results showed that the variation range of the benthic biodiversity index was 1.00–2.73, with an average of 2.00. There were 31 species, 27 families, and 8 phyla in the autumn survey. Among them, the number of annelid species was relatively large, and the composition of biomass was larger in annelid and echinoderms, followed by arthropods and mollusks. The results of autumn survey showed that the variation range of the benthic biodiversity index in the sea area was 1.00–2.50, with an average of 1.83.

### 3.3. Interrelation and Source Analysis of Heavy Metals

**Figure 6:**
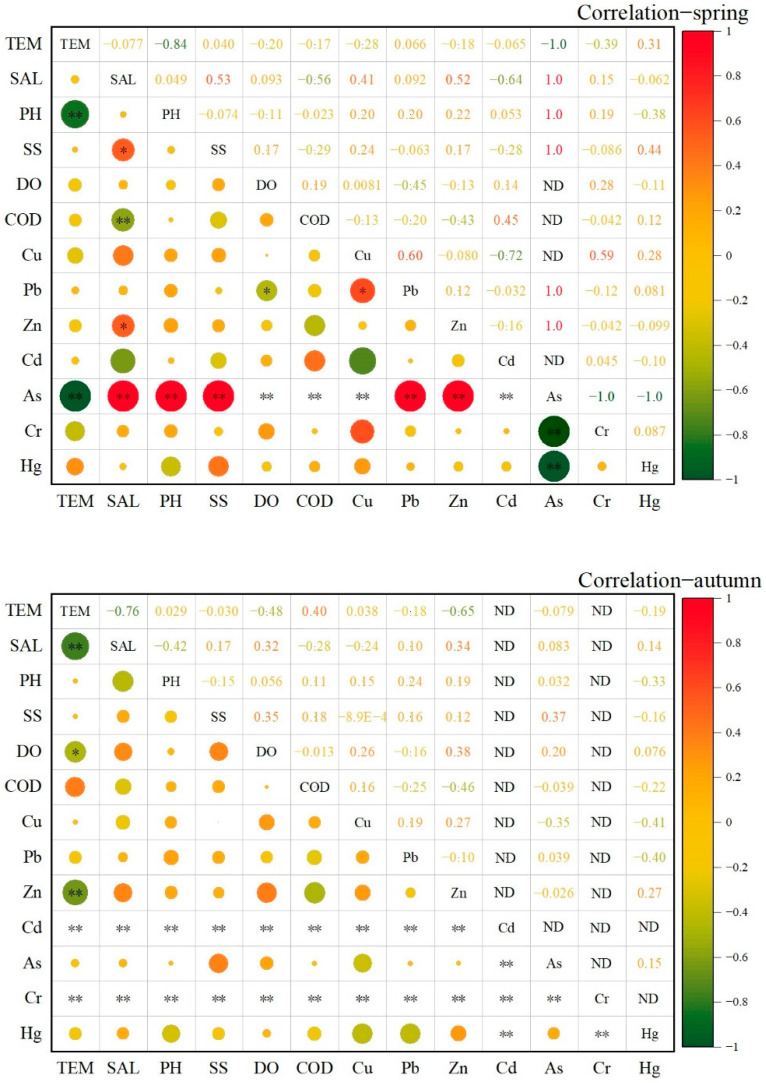
The correlational analysis of heavy metals in the seawater of coastal Hong Kong (* *p* < 0.05; ** *p* < 0.01).

The results of correlational analysis between heavy metals and other particulate matters in the coastal waters of Hong Kong are shown in Figure 6. According to the correlational matrix, pH was positively correlated with the other, metals except Hg, indicating that the change in heavy metal concentration was likely to be the main influencing factor of pH change. Salinity was negatively correlated with Cd and Hg in spring, and negatively correlated with Cu in autumn, indicating that salinity may hinder seawater mixing and maintain high concentrations of heavy metals in seawater. The correlation between Pb and Cu was as high as 0.6 in spring and 0.19 in autumn, indicating that the two heavy metals were likely to have the same source, but seasonal changes in production activities lead to seasonal changes in different heavy metal contents. The correlation between Zn and Cd and other heavy metals was negative, indicating that Zn and Cd may be coming from a different source than the emission sources of the other heavy metals. Due to the lower detection of As in spring water samples and Cr and Cd in autumn water samples, the correlational analysis results of Cr and Cd in autumn were all zero. Cheung and Wong (1992) also showed that the As content in the ocean was very low compared with Cu, Cd, and Cr, and there was no specific As pollution source in Hong Kong, because As usually comes from natural sources [39].

*K_d_* can be used to describe the adsorption capacity of heavy metals from seawater to sediment, which is affected by the characteristics of heavy metals and changes in sediment and seawater. The *K_d_* results near the coast of Hong Kong are shown in Table 2. As no Cd was detected in the sediments, the pollution of Cd was very low in the sediments near the coast of Hong Kong, and this evaluation was not carried out. In this study, in addition to Cd, the order of *K_d_* values in spring and autumn were Cr > Pb > Cu > Zn > As > Hg. The *K_d_* values of Cr and Pb were much higher than those of the other heavy metal elements, indicating that, in the seawater environment, sediments are easy to enrich with Cr and Pb from seawater and allow them to accumulate. In terms of time, the *K_d_* value in spring was greater than that in autumn, indicating that the adsorption capacity of heavy metals by sediments from seawater is affected by a certain temperature, which is also related to the different concentrations of heavy metals in seawater in different seasons. In terms of spatial distribution, the distribution trends of heavy metals were similar, and the *K_d_* value in the southeast of the study area was much higher than that in the northwest coast, indicating that heavy metals are more likely to be enriched by sediments in deep water areas far from the coast.

Since the reform and opening up in 1978, the Pearl River has been polluted by heavy metals. After decades of accumulation, the Pearl River has accumulated large amounts of heavy metals in seawater, sediments, and organisms [40,41,42]. The water of Pearl River is polluted by metal processing and electroplating industries, as well as industrial wastewater and domestic sewage discharge [43], resulting in an increase in heavy metals such as Pb, Zn, and Cu discharged into the ocean. After the Pearl River enters the sea, it spreads to the offshore coast of Hong Kong, resulting in an increase in heavy metal concentrations. Industrial and municipal sewage discharge and maritime shipping are the main sources of Zn, Pb, Cu, and Cd; hence, they are greatly affected by human activities. Due to the large population of Hong Kong and well-developed high-tech industry, it is inevitable that heavy metals will accumulate in coastal seawater, sediments, and organisms. Compared with the other heavy metals, the enrichment degree of Cu was higher, which may be due to serious pollution discharge in electroplating, electronic circuit board production, alloy smelting, textile, and other industries in Kwun Tong and Tsuen Wan, two important old industrial bases in Hong Kong. The source of As may be from aquaculture off the coast of Hong Kong.

### 3.4. Ecological Risk Assessment

**Figure 7:**
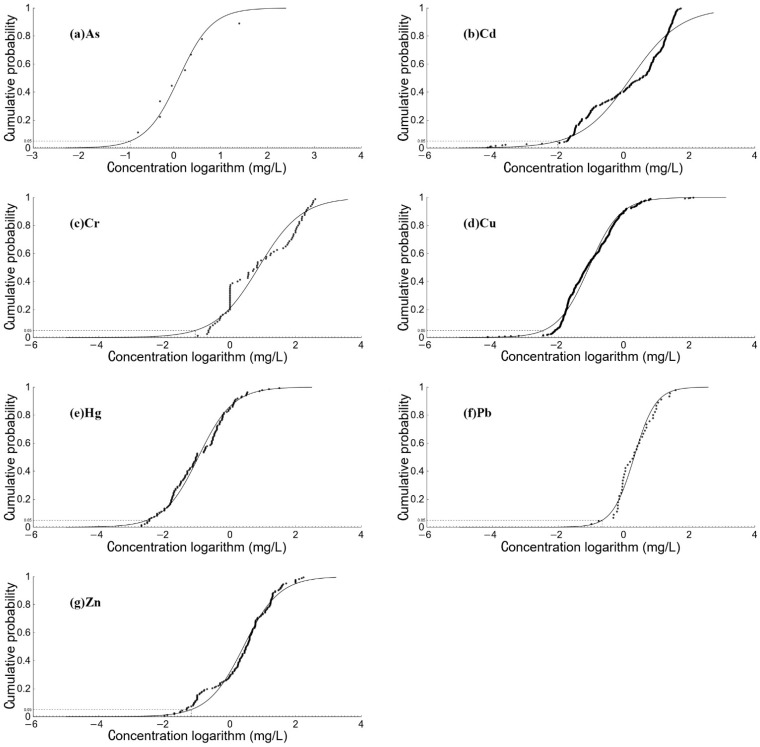
The species sensitivity distribution of heavy metals in coast of Hong Kong.

The potential biological hazard effects of heavy metals in the coastal waters of Hong Kong obtained by the species sensitivity distribution method are shown in Figure 7 and Table S6. In terms of the types of heavy metals, Cu and Zn were the main factors causing ecological risks, and the other heavy metals did not harm organisms in the sea area. In terms of spatial distribution, the potential hazard ratio at sites close to the coast in the west and north was significantly higher than at sites far from the coast, especially for Cu detected in the spring water samples, which may be related to pollutant emissions and hydrodynamic conditions in Hong Kong ‘s high-tech industries [44,45]. As mentioned earlier, the industrial structure of the two old industrial bases in Hong Kong, which includes the production of electroplating, electronic circuit boards, and smelting of alloys, are bound to generate a lot of heavy metal pollution. Mangroves in Hong Kong also had a strong capacity to retain heavy metals, which means that even if local governments strengthen pollution control and move industries to mainland China, heavy metal pollution in Hong Kong will still produce certain ecological risks.

**Figure 8:**
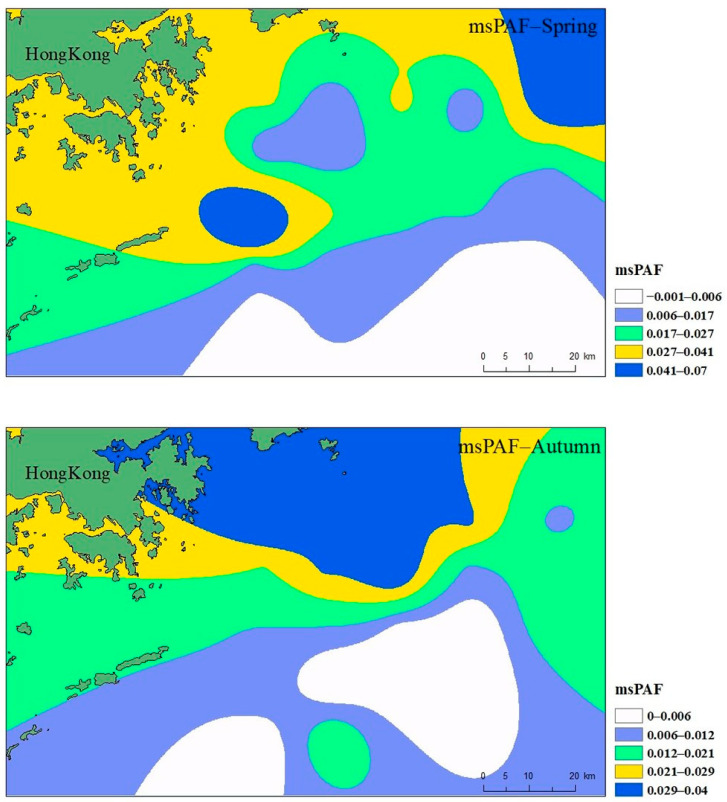
Compound hazard ratio (msPAF) of heavy metals in seawater of Hong Kong.

The spatial distribution interpolation of the compound hazard ratio on the coast of Hong Kong is shown in Figure 8. It can be seen from Figure 8 that the joint ecological risk spatial distribution of multiple heavy metals in the coastal waters of Hong Kong has obvious directivity, and gradually decreases from the near point to the far point along the Hong Kong coast. The autumn map shows that the combined ecological risk of various heavy metals gradually decreases from north to south, and the highest value appears near the coast of Shenzhen. Shenzhen is a typical city of China that has rapidly urbanized in the past 40 years. The ecological risk of the Daya Bay nuclear power offshore area in the east was higher, which may have been caused by marine transportation. This area was the berth of cargo ships transporting petrochemical products. The ballast water or sewage from cargo ships can increase heavy metal content [38]. At the same time, Hong Kong is in the export of the Pearl River and adjacent to Shenzhen. The Pearl River estuary region collects a lot of industrial, agricultural, and ship sewage [46]. Shenzhen has many electronic, communication, and metal manufacturing industries, and accumulates several times the amount of heavy metal pollution emitted by the processing industry in the marine sediments of the two places. This may be the main reason why the biological sensitivity risk of heavy metals in Hong Kong offshore is significantly higher than that of other regions. Therefore, effective wastewater treatment of the precision instrument processing industry and high-tech industry in Hong Kong and its surrounding areas would be an effective measure to reduce the ecological risk of the region.

### 3.5. Priority Control and Recommendations

Heavy metals in the ocean are important factors affecting the ecological environment, aquatic organisms, and even human health. Understanding the distribution of different heavy metals is essential for local governments to properly manage and protect the marine environment. Although there is a certain degree of heavy metal pollution in the coastal waters of Hong Kong, the overall pollution level is clean. The contents of Cu and Zn in seawater were higher, and the heavy metals in organisms detected in spring were higher than in autumn. This may indicate that organisms near the coast are easy to be enriched by oxidants in cold seasons, which explains why many organisms, especially invertebrates, have higher biochemical reactions in cold seasons. Therefore, the investigation of heavy metals along the coast of Hong Kong is important, not only to provide accurate information for the precise control of heavy metal pollution and avoid wasting resources, but it also enables the aquatic product industry to further improve the quality of aquatic products by avoiding polluted areas.

The pollution of human activities in Hong Kong was the most serious in the Victoria Bay in the north of Hong Kong Island and Tulu Port in the northeast. The pollution caused by domestic sewage, industrial wastewater, agricultural surface runoff, and shipping was mainly concentrated in these two areas. To ensure environmental quality, the focus of heavy metal pollution detection should be to the northeast of Hong Kong and the coastal areas. At the same time, heavy metals that are harmful to the environment should also be considered. This would not only optimize the monitoring network of heavy metals, but also improve monitoring efficiency and help obtain more accurate environmental quality data.

We found that the accumulation effects of heavy metals in seawater, sediments, and organisms were different due to the different types of heavy metals. If a single evaluation method is used, the pollution level of heavy metals cannot be well-evaluated. In this study, the ecological distribution characteristics of heavy metals, the migration level between different media and the evaluation system of species sensitivity distribution were established to effectively evaluate the accumulation effects of heavy metals in seawater, sediments, and organisms. The results of this study would provide sufficient information to support coastal ecological restoration in Hong Kong. As an important part of the Guangdong-Hong Kong-Macao Greater Bay Area, Hong Kong should pay attention to the protection of its marine environment while developing its economy, which requires Hong Kong to plan in advance for sustainable and high-quality development suited to its specific geographical environment and industrial layout.

## 4. Conclusions

In this study, the heavy metal pollution in the offshore of Hong Kong was evaluated by a variety of methods. The results showed that the pollution level of heavy metals in seawater and sediments in the offshore of Hong Kong was not high, and the degree of heavy metal pollution was low. In spring, the heavy metals in marine organisms exceeded the standard, especially in coastal areas north of Hong Kong. The distribution of heavy metal concentrations in the waters off Hong Kong also showed some seasonal variation, which was particularly evident for Cu, Pb, and Cr. This may be due to the uneven distribution of seawater caused by the seasonal monsoon in Hong Kong. Through the analysis, we found that heavy metal pollution near the coast of Hong Kong was closely related to the precision instrument processing industry, domestic sewage, industrial wastewater, and shipping. The correlational analysis of heavy metals showed that Zn and Cd had negative correlations with several other heavy metals, which may indicate that they come from different emission sources; the correlation between Pb and Cu has a larger difference in spring and autumn, which may be due to seasonal changes in production activities. SSD biological evaluation showed that Cu and Zn were the main factors causing ecological risk, and the risk gradually decreased from north to south.

The heavy metal risk distribution and SSD distribution obtained in this study can enable decision-makers to formulate efficient and well-founded pollution-control measures and develop them in concert with the production of marine aquatic products. The northwest and northeast regions of Hong Kong are more likely to be considered polluted. One of these two regions was the estuary of the Pearl River, and the other was the coast of Shenzhen. Therefore, decision-makers should strengthen supervision and governance of these two locations and strictly control the discharge of sewage. Future research should also focus on prevention and control to better protect marine ecosystems.

## Figures and Tables

**Table 1:** Results of single pollution index evaluation.

Carrier	Season	Statistical Parameter	Cu	Pb	Zn	Cd	As	Cr	Hg
Seawater	Spring	Max	0.96	1.19	1.29	0.09	0.35	0.01	1.2
		Min	0	0.02	0	0	0	0	0
		CV	0	9.5	19	0	0	0	0
	Autumn	Max	0.36	3.62	1.35	0.02	0.06	0.01	2.36
		Min	0	0	0	0	0.04	0	0.34
		CV	0	14.3	14.3	0	0	0	33.3
Marine organism	Spring	Max	0.13	4.3	1.81	14.4	4.5	5.17	0.21
		Min	0.01	0.06	0.07	0.03	2.11	0.09	0.02
		CV	0	23.08	23.08	26.92	26.92	26.92	0
	Autumn	Max	0.33	0.03	0.21	0.73	0	0.65	0.09
		Min	0	0.03	0.04	0.01	0	0.16	0.02
		CV	0	0	0	0	0	0	0
Sediments	Spring	Max	0.32	0.65	0.53	0	0.44	0.47	0.27
		Min	0.09	0.25	0.35	0	0.18	0.35	0.17
		CV	0	0	0	0	0	0	0

The seawater index was judged according to the People’s Republic of China Seawater Quality Standards (GB3097-1997); the sediment index was judged according to the Specifications for Oceanographic Survey (GB/T 12763-2007); the marine organism index was judged according to the Countrywide Comprehensive Investigations of the Coastal Zone and Tidal Land Resources and Technical Regulations for the Second National Baseline Survey of Marine Pollution.

**Table 2:** Flowability of heavy metals in coastal Hong Kong.

Season	Spring							Autumn						
Site	Cu	Pb	Zn	Cd	As	Cr	Hg	Cu	Pb	Zn	Cd	As	Cr	Hg
H1	8.23	141.00	6.55	0.00	0.00	150.80	1.75	5.94	51.27	3.74	0.00	10.83	0.00	2.33
H2	0.00	142.11	13.57	0.00	0.00	310.00	2.05	9.20	7.46	8.34	0.00	6.77	0.00	1.95
H3	1.55	25.53	4.22	0.00	0.00	76.67	0.00	0.00	71.85	6.18	0.00	5.93	0.00	0.95
H5	0.00	1075.00	0.00	0.00	0.00	125.56	1.57	0.00	97.73	0.00	0.00	4.74	0.00	0.66
H6	0.00	123.75	4.99	0.00	0.00	201.76	1.15	11.20	11.34	4.56	0.00	9.76	0.00	1.74
H8	16.50	91.25	3.03	0.00	0.89	182.00	1.60	0.00	42.12	14.07	0.00	7.76	0.00	1.17
H9	0.00	89.44	7.01	0.00	0.00	262.14	1.16	0.00	0.00	0.00	0.00	4.63	0.00	1.16
H10	0.00	82.22	8.82	0.00	4.93	82.82	0.95	11.17	82.22	6.31	0.00	3.95	0.00	1.50
H12	0.00	75.76	3.85	0.00	0.00	255.00	2.80	5.63	39.68	4.02	0.00	5.73	0.00	1.91
H13	32.17	68.97	2.44	0.00	0.00	125.38	3.00	0.00	72.70	18.56	0.00	5.58	0.00	1.14
H15	0.00	162.92	6.97	0.00	0.00	69.56	0.00	0.00	391.00	2.49	0.00	6.60	0.00	0.45
H17	0.00	28.32	2.63	0.00	0.00	198.00	0.00	0.00	120.36	15.29	0.00	4.88	0.00	1.00
H18	27.50	195.38	18.47	0.00	0.00	195.00	1.18	0.00	97.69	0.00	0.00	2.97	0.00	0.81
H19	1.26	25.43	4.78	0.00	0.00	177.65	1.21	9.40	23.23	4.14	0.00	4.89	0.00	2.41
H21	15.71	62.56	4.15	0.00	0.00	153.89	4.00	5.50	87.14	6.48	0.00	4.21	55.40	0.94

## Supplementary Materials

The following supporting information can be downloaded at: https://www.mdpi.com/article/10.3390/ijerph192013376/s1.
