# Supplementary material for: Comprehensive Ecological Risk Assessment of Heavy Metals Based on Species Sensitivity Distribution in Aquatic of Coastal Areas in Hong Kong
Source: Int J Environ Res Public Health. 2022 Oct 17;19(20):13376. doi: 10.3390/ijerph192013376 (PMC9602570; doi:10.3390/ijerph192013376)
Supplement: Supplementary file 1 [file ijerph-19-13376-s001.zip › ijerph-1923470-supplementary.pdf]

Table S1 Detection limits and relative standard deviation of seven heavy metals in seawater, sediments, and organisms.

		Hg	Cd	Pb	Cu	Cr	As	Zn
Detection limit	Seawater (μg/L)	0.001	0.010	0.030	0.200	0.400	0.500	3.100
	Sediments (mg/kg)	0.002	0.040	1.000	0.500	2.000	0.060	6.000
	Organisms (mg/kg)	0.002	0.005	0.040	0.400	0.040	0.200	0.400
Relative standard deviation	Seawater	2.50%	4.20%	3.90%	5.20%	3.20%	3.00%	6.90%
	Sediments	4.00%	6.60%	6.30%	0.10%	2.90%	5.00%	2.30%
	Organisms	8.00%	3.60%	6.40%	1.60%	10.00%	1.50%	4.90%

Table S2 The raw data of seven heavy metals in seawater.

Site	Cu	Pb	Zn	Cd	As	Cr	Hg	Cu	Pb	Zn	Cd	As	Cr	Hg
	(μg/L)							(μg/L)						
Season	Spring							Autumn						
H1	1.3	0.2	11.26	0.01	ND	0.25	0.028	1.8	0.55	19.7	0.02	0.8	ND	0.021
H2	ND	0.19	5.04	0.01	ND	0.11	0.02	1	3.62	8.2	ND	1.2	ND	0.021
H3	4.78	0.76	15.25	0.02	ND	0.45	ND	ND	0.27	10.4	0.02	1	ND	0.041
H4	0.25	0.47	16.46	0.09	ND	0.26	0.022	0.6	0.13	ND	ND	1	ND	0.076
H5	ND	0.02	ND	ND	ND	0.27	0.021	ND	0.22	ND	ND	1	ND	0.05
H6	ND	0.24	15.9	ND	ND	0.17	0.047	1	2.62	17.4	0.02	0.9	ND	0.031
H7	ND	0.26	12.03	ND	ND	0.14	0.021	0.7	0.2	19.4	0.02	1.2	ND	0.023
H8	0.6	0.24	25.11	ND	6.94	0.2	0.03	ND	0.52	5.4	ND	0.8	ND	0.041
H9	ND	0.18	9.95	ND	ND	0.14	0.031	ND	ND	ND	ND	1	ND	0.031
H10	ND	0.18	7.52	ND	0.88	0.39	0.038	0.6	0.18	10.5	ND	1.1	ND	0.024
H11	1.26	0.95	11.71	0.04	ND	0.15	0.047	0.6	0.2	23.6	0.02	1.1	ND	0.055
H12	ND	0.33	18.38	ND	ND	0.14	0.015	1.6	0.63	17.6	0.02	1.2	ND	0.022
H13	0.23	0.39	25.87	ND	ND	0.26	0.016	ND	0.37	3.4	ND	1.2	ND	0.042
H14	0.34	0.84	5.88	0.09	ND	0.18	ND	ND	0.21	ND	ND	1.1	ND	0.037
H15	ND	0.24	9.66	ND	ND	0.45	ND	ND	0.1	27	ND	1	ND	0.093
H16	ND	0.17	18.93	ND	ND	0.24	0.06	0.6	0.11	ND	ND	1.2	ND	0.118
H17	ND	1.19	22.11	0.01	ND	0.15	ND	ND	0.28	3.8	ND	0.9	ND	0.034
H18	0.2	0.13	3.8	ND	ND	0.18	0.044	ND	0.26	ND	ND	1.2	ND	0.064
H19	3.74	1.16	12.83	0.01	ND	0.17	0.034	0.5	1.27	14.8	ND	1.1	ND	0.017
H20	0.3	0.29	23	ND	ND	0.16	0.036	ND	ND	22.2	ND	0.9	/	0.029
H21	0.21	0.39	12.79	ND	ND	0.18	0.012	0.6	0.28	8.2	ND	1	0.5	0.051
ND indicates not detected.														

Table S3 The raw data of seven heavy metals in sediment.

Site	Cu	Pb	Zn	Cd	As	Cr	Hg
	mg/kg						
H1	10.7	28.2	73.7	ND	8.66	37.7	0.049
H2	9.2	27	68.4	ND	8.12	34.1	0.041
H3	7.4	19.4	64.3	ND	5.93	34.5	0.039
H5	7.7	21.5	66	ND	4.74	33.9	0.033
H6	11.2	29.7	79.3	ND	8.78	34.3	0.054
H8	9.9	21.9	76	ND	6.21	36.4	0.048
H9	9	16.1	69.7	ND	4.63	36.7	0.036
H10	6.7	14.8	66.3	ND	4.34	32.3	0.036
H12	9	25	70.7	ND	6.88	35.7	0.042
H13	7.4	26.9	63.1	ND	6.69	32.6	0.048
H15	6.6	39.1	67.3	ND	6.6	31.3	0.042
H17	4.6	33.7	58.1	ND	4.39	29.7	0.034
H18	5.5	25.4	70.2	ND	3.56	35.1	0.052
H19	4.7	29.5	61.3	ND	5.38	30.2	0.041
H21	3.3	24.4	53.1	ND	4.21	27.7	0.048
ND indicates not detected.							

Table S4 The raw data of seven heavy metals in organisms.

Site	Season	Species	Cu	Pb	Zn	Cd	Hg	As	Cr
y1	Spring	Greater lizardfish	<0.4	0.22	7	0.049	0.022	1.17	1.54
y2		Greater lizardfish	<0.4	0.12	4.4	0.045	0.012	1.43	0.51
y3		Greater lizardfish	<0.4	0.29	4.4	0.046	0.031	0.94	2.36
y4		Greater lizardfish	<0.4	0.41	5.6	0.044	0.006	1.98	2.7
y5		Greater lizardfish	<0.4	0.3	4.8	0.052	0.011	2.86	7.03
y6		Greater lizardfish	<0.4	0.22	5.2	0.034	0.024	1.57	0.58
y7		Saurida elongata	0.5	0.16	4.7	0.028	0.006	1.36	0.38
y8		Greater lizardfish	<0.4	0.15	4.8	0.047	0.005	1.17	0.92
y9		Greater lizardfish	<0.4	0.24	7.2	0.054	<0.002	0.93	6.27
y10		Greater lizardfish	<0.4	0.31	5.7	0.019	0.015	1.45	0.38
y11		Saurida elongata	<0.4	0.32	5.3	0.053	0.018	0.86	0.28
y12		Saurida elongata	<0.4	0.53	7.1	0.067	0.005	1.15	7.75
y1		Charybdis miles	4.9	0.36	24	1.37	0.009	16.8	0.33
y2		Amussium japonicum formosum	0.8	0.36	26.4	2.1	0.008	3	0.11
y3		Charybdis granulata	8.1	0.32	24.9	0.648	0.041	25.5	1.03
y4		White-hair rough shrimp	1.5	0.34	12.3	0.08	0.009	7.74	0.32
y5		White-hair rough shrimp	1.4	0.43	11.2	0.066	0.006	6.27	5.09
y5		Amussium japonicum formosum	<0.4	0.37	27.2	0.777	0.008	4.5	0.05
y6		White-hair rough shrimp	1.2	0.37	12.9	0.054	0.02	8.12	0.3
y7		White-hair rough shrimp	2	0.36	13.5	0.054	0.034	7.9	0.13
y7		Amussium japonicum formosum	1	0.39	22.9	1.14	0.005	3	0.06
y8		Amussium japonicum formosum	0.7	0.32	26.8	2.05	<0.002	2.61	0.05
y10		Amussium japonicum formosum	0.5	0.25	17.5	0.564	<0.002	2.11	0.06
y11		Calappa philargius	4.8	0.23	57.4	1.28	0.037	37.9	1.31

y11		Amussium japonicum formosum	0.5	0.34	21.4	2.88	<0.002	2.58	0.07
y12		Amussium japonicum formosum	1.3	0.43	36.2	0.769	<0.002	4.18	0.19
y1	Autumn	Decapterus maruadsi	0.07	<0.04	8.52	0.013	0.016	0.9	0.69
y2		Blood porgy	<0.4	<0.04	5.85	0.007	0.017	1.4	0.64
y3		White croaker	<0.4	<0.04	3.98	<0.005	0.013	0.8	0.47
y4		Blood porgy	<0.4	<0.04	6.76	<0.005	0.014	1.6	0.66
y5		White croaker	<0.4	<0.04	4.55	0.006	0.006	0.5	0.5
y6		White croaker	<0.4	0.06	4.04	<0.005	0.011	0.5	0.55
y7		White croaker	0.4	<0.04	4.18	<0.005	0.01	1.8	0.26
y8		White croaker	<0.4	<0.04	4.39	<0.005	0.008	0.9	0.43
y9		White croaker	<0.4	<0.04	4.02	<0.005	0.008	0.6	0.47
y10		Nemipterus japonicus	<0.4	<0.04	4.07	<0.005	0.005	1	0.97
y11		White croaker	<0.4	<0.04	3.46	0.006	0.01	0.5	0.24
y12		White croaker	<0.4	0.05	4.38	<0.005	<0.002	0.7	0.32
y1		Metapenaeus affinis	4.4	<0.04	12.8	0.013	0.013	6.4	0.79
y2		Bursa elegans	7.5	<0.04	9.39	0.596	0.01	6	0.8
y3		Squillid	21.6	<0.04	15.3	1.46	0.007	11	0.43
y4		Squillid	24.8	<0.04	12.8	1.17	0.005	7.9	0.3
y5		Squillid	13	<0.04	15.8	1.26	0.013	6.5	0.56
y6		Squillid	10.3	<0.04	17.9	1.25	0.017	2.8	0.48
y7		Squillid	12.6	<0.04	14	0.556	0.013	5.7	0.73
y8		Squillid	11.5	<0.04	14.5	0.575	0.018	4.7	0.38
y9		Portunus sanguinolentus	17.7	<0.04	17.6	0.27	0.012	3.8	0.76
y10		Squillid	16	<0.04	13.8	0.773	0.012	3.6	0.57
y12		Metapenaeopsis palmensis	33.4	<0.04	11.2	0.012	0.011	6.2	0.85

Table S5 Evaluation results of zooplankton biodiversity index.

Site	Spring			Autumn		
	Species	total individuals	Diversity Index H'	Species	total individuals	Diversity Index H'
H1	20	132	3.96	22	246	3.94
H2	21	203	3.9	22	612	2.34
H3	18	149	3.38	19	563	3.56
H5	15	253	3.21	21	234	3.99
H6	15	187	3.37			
H8	19	300	3.59	18	270	3.59
H9	17	277	3.62	30	439	4.15
H10	16	196	3.55	20	358	3.65
H12	15	207	3.61	20	231	3.65
H13	23	421	3.97	19	268	3.72
H15	14	182	3.46	18	387	3.46
H17	18	267	3.7			
H18	20	435	2.48	20	684	3.66
H19	13	174	3.44	16	164	3.55
H21	18	267	3.7	18	140	3.76
Mean	17	243	3.53	20	353	3.62
Range	13~23	132~435	2.48~3.97	16~30	140~684	2.34~4.15

Table S6 Potential hazard ratio (PAF) in seawater of HongKong (%).

Time	Research stance	Cu	Pd	Zn	Cd	As	Cr	Hg
Spring	H1	2	0	1	0	0	0	0
	H2	0	0	1	0	0	0	0
	H3	6	0	1	0	0	0	0
	H4	0	0	1	0	0	0	0
	H5	0	0	0	0	0	0	0
	H6	0	0	1	0	0	0	0
	H7	0	0	1	0	0	0	0
	H8	1	0	2	0	0	0	0
	H9	0	0	1	0	0	0	0
	H10	0	0	1	0	0	0	0
	H11	2	0	1	0	0	0	0
	H12	0	0	2	0	0	0	0
	H13	0	0	2	0	0	0	0
	H14	0	0	1	0	0	0	0
	H15	0	0	1	0	0	0	0
	H16	0	0	2	0	0	0	0
	H17	0	0	2	0	0	0	0
	H18	0	0	0	0	0	0	0
	H19	5	0	1	0	0	0	0
	H20	0	0	2	0	0	0	0
	H21	0	0	1	0	0	0	0
Autumn	H1	2	0	2	0	0	0	0

	H2	1	0	1	0	0	0	0
	H3	0	0	1	0	0	0	0
	H4	1	0	0	0	0	0	0
	H5	0	0	0	0	0	0	0
	H6	1	0	2	0	0	0	0
	H7	1	0	2	0	0	0	0
	H8	0	0	1	0	0	0	0
	H9	0	0	0	0	0	0	0
	H10	1	0	1	0	0	0	0
	H11	1	0	2	0	0	0	0
	H12	2	0	2	0	0	0	0
	H13	0	0	0	0	0	0	0
	H14	0	0	0	0	0	0	0
	H15	0	0	3	0	0	0	0
	H16	1	0	0	0	0	0	0
	H17	0	0	0	0	0	0	0
	H18	0	0	0	0	0	0	0
	H19	0	0	1	0	0	0	0
	H20	0	0	2	0	0	0	0
	H21	1	0	1	0	0	0	0
